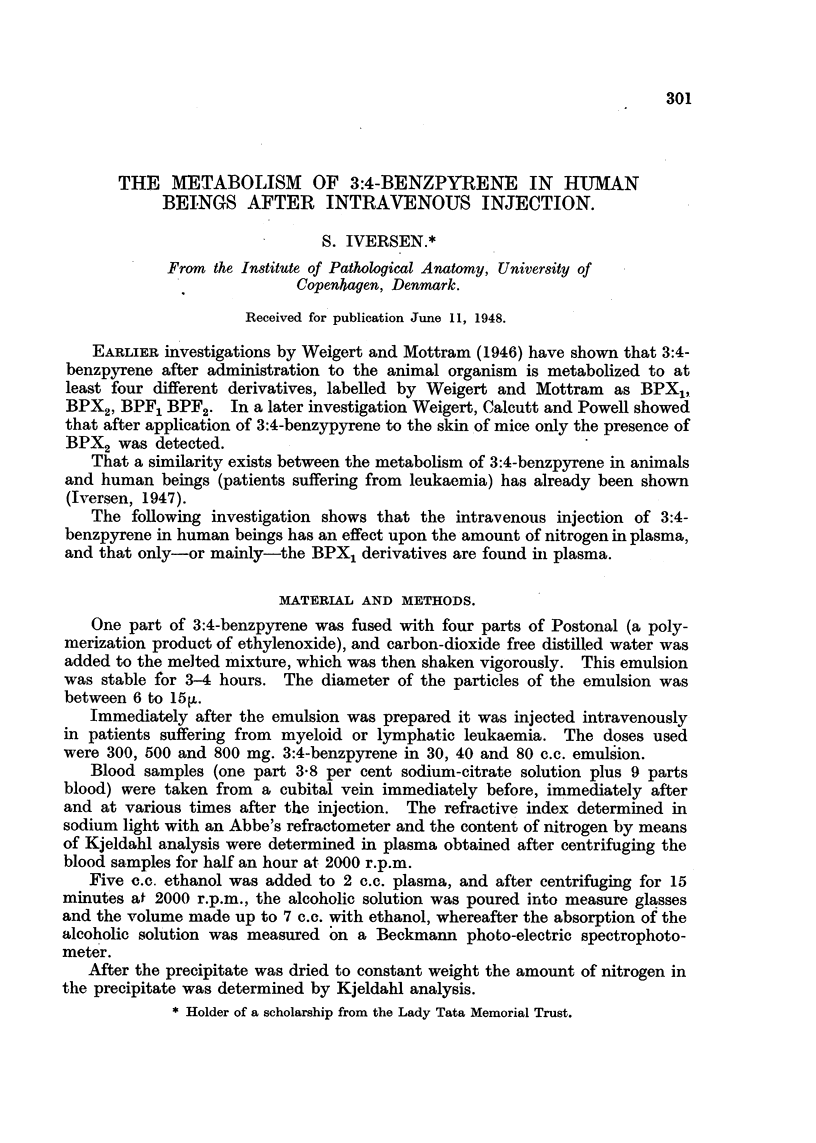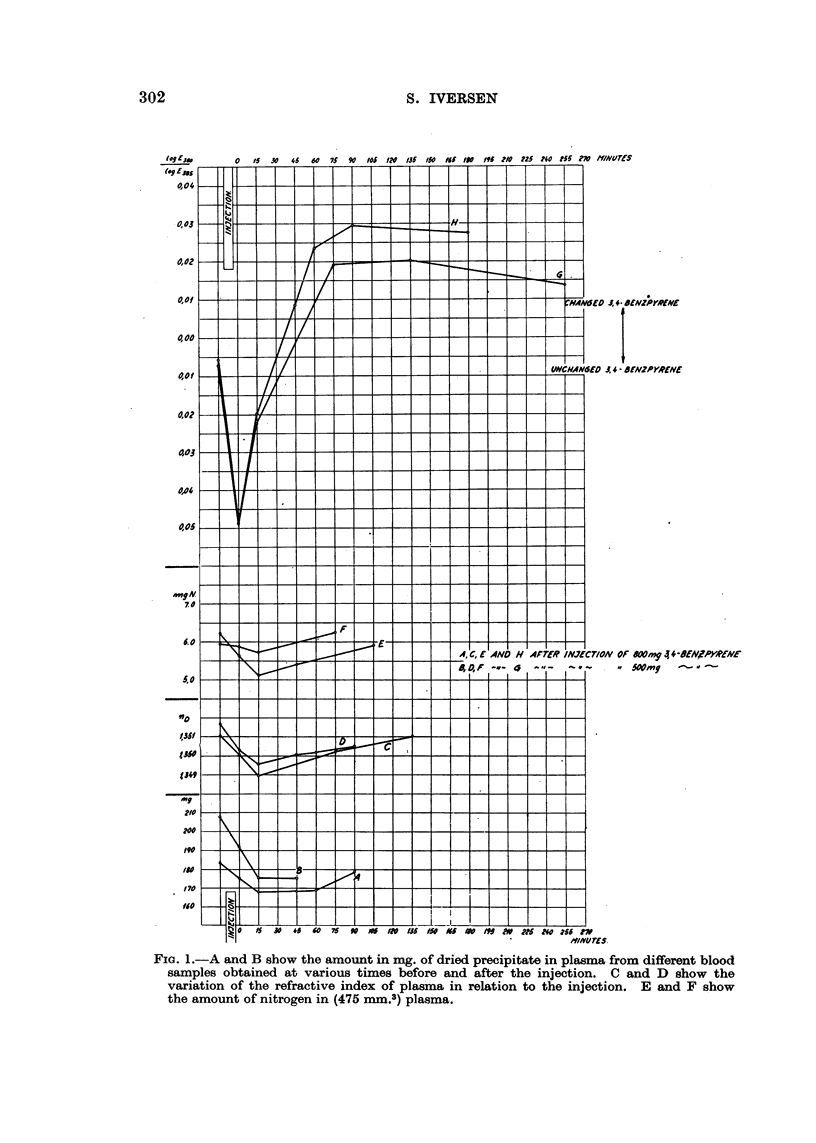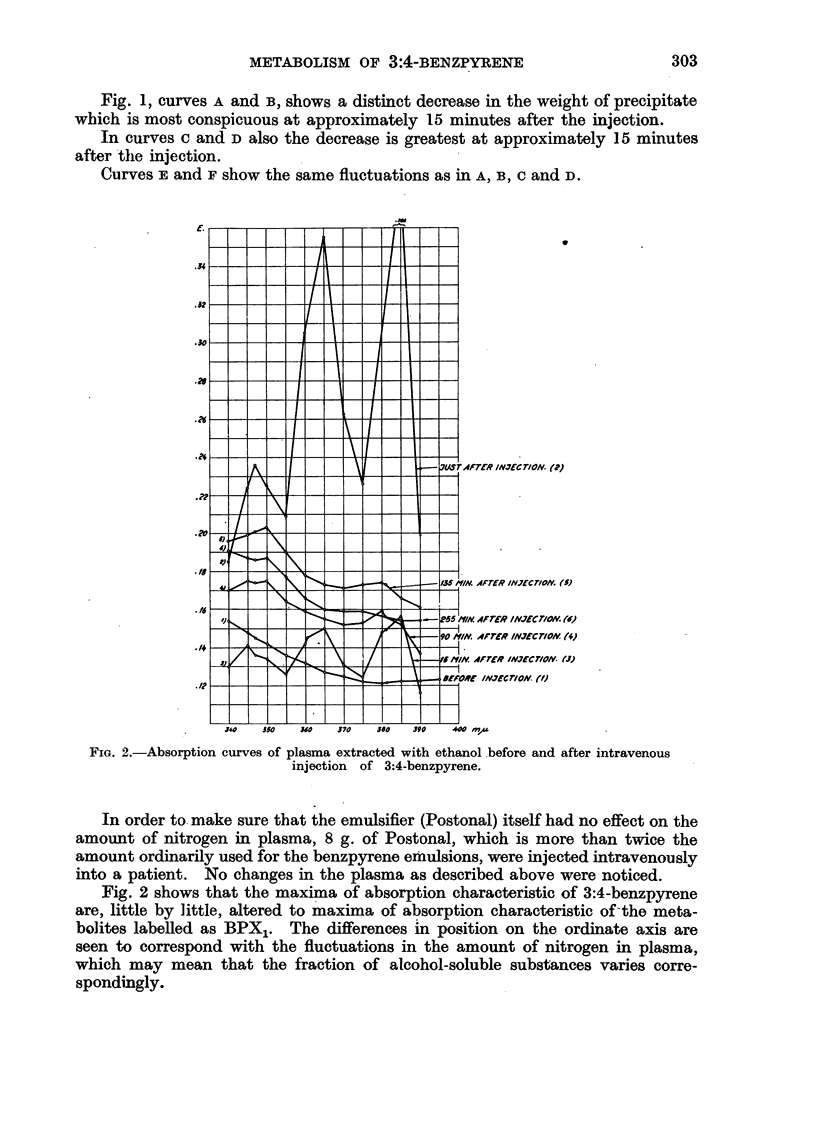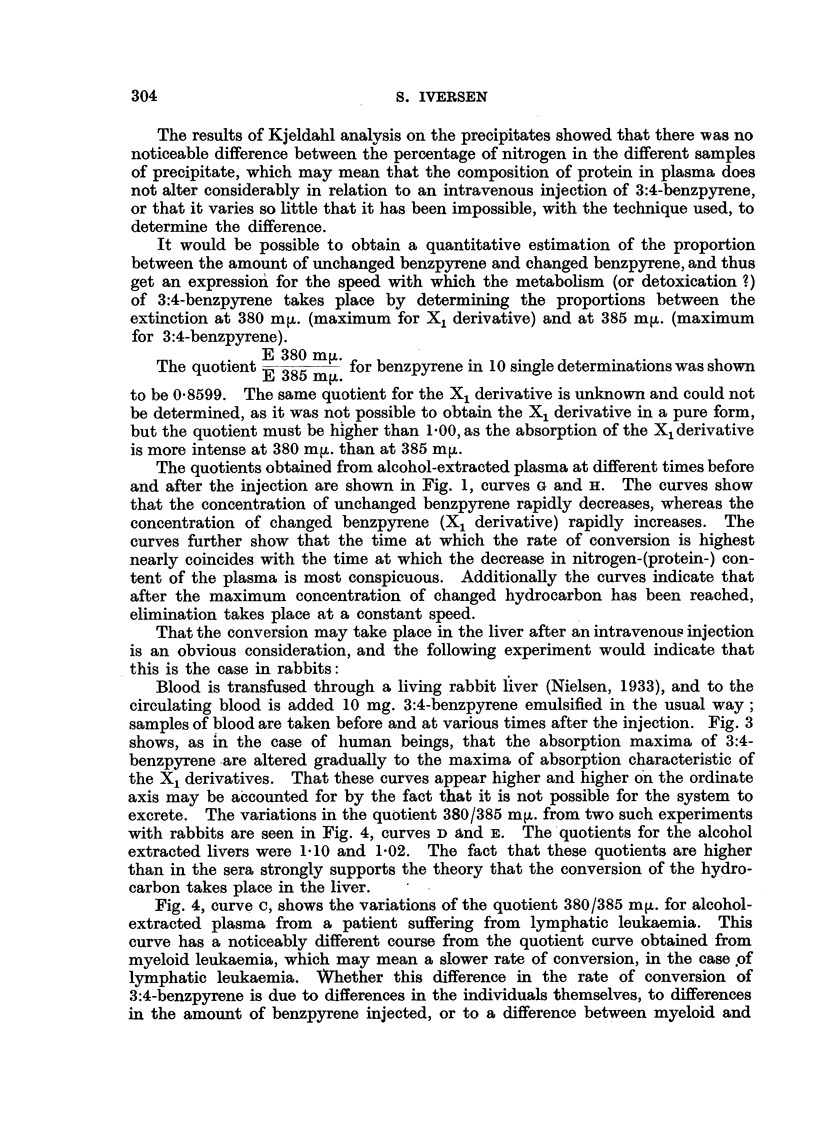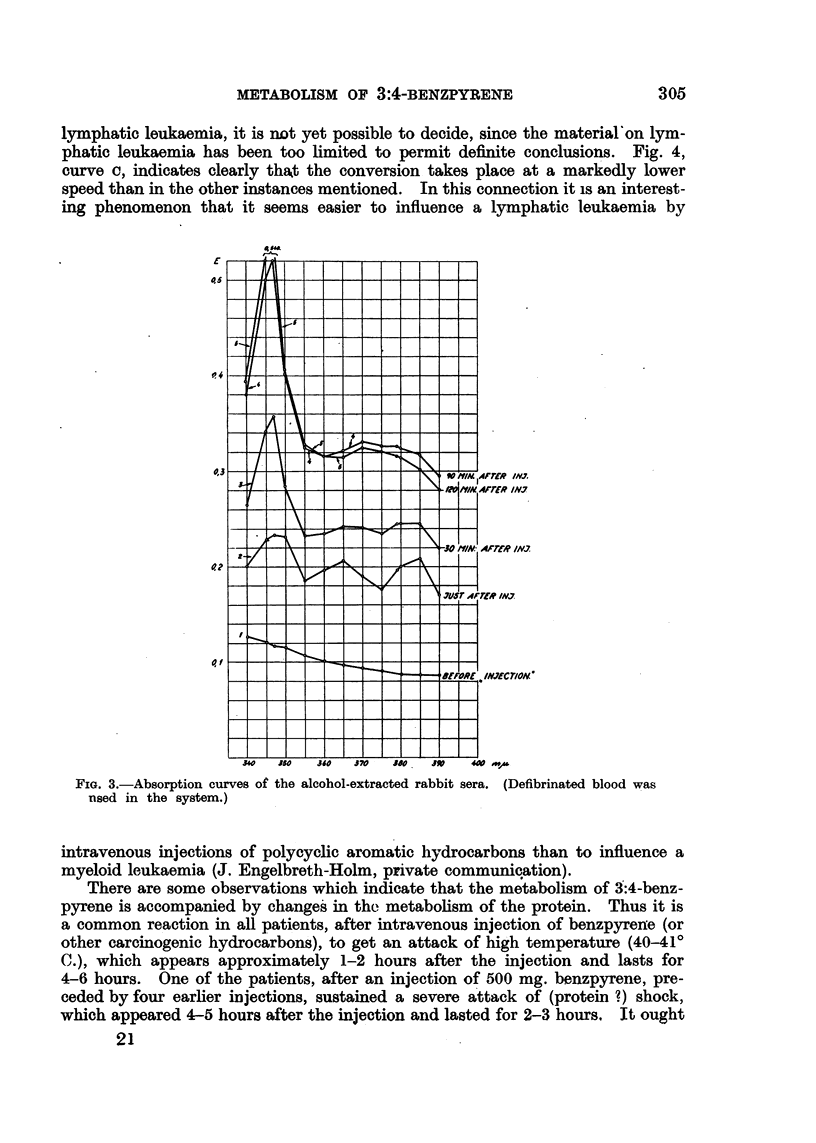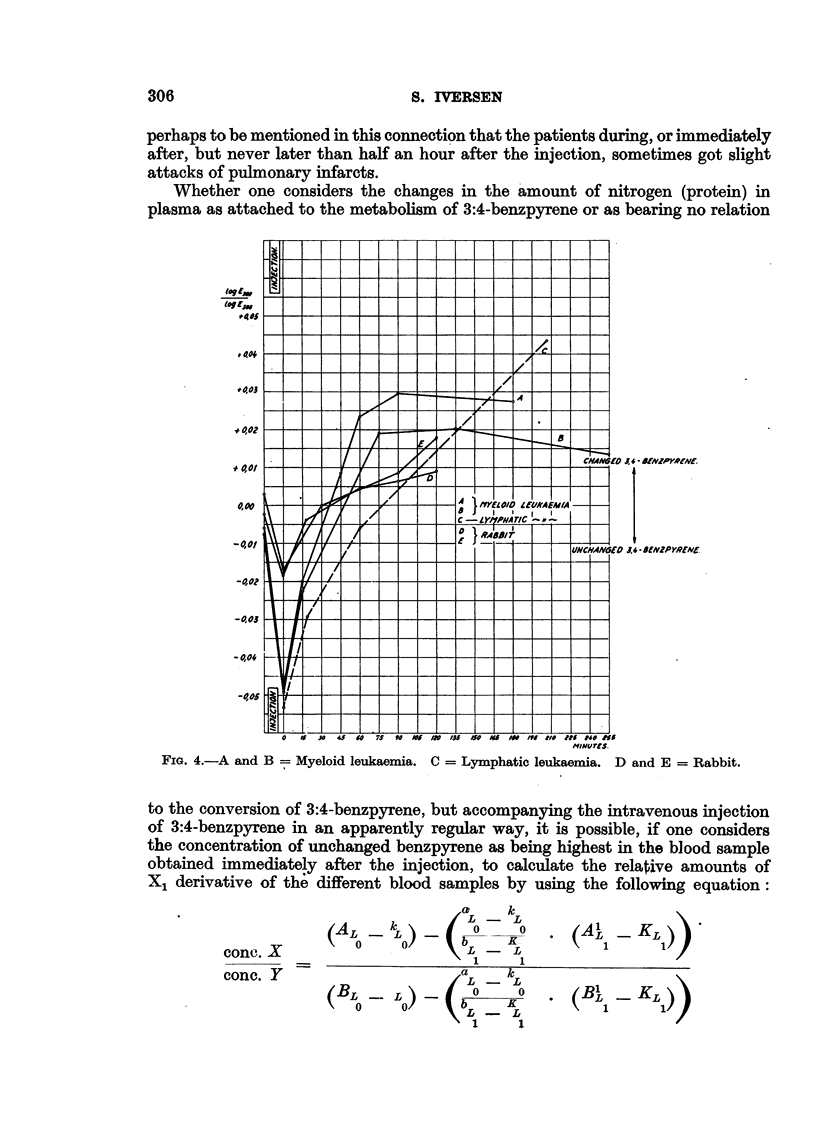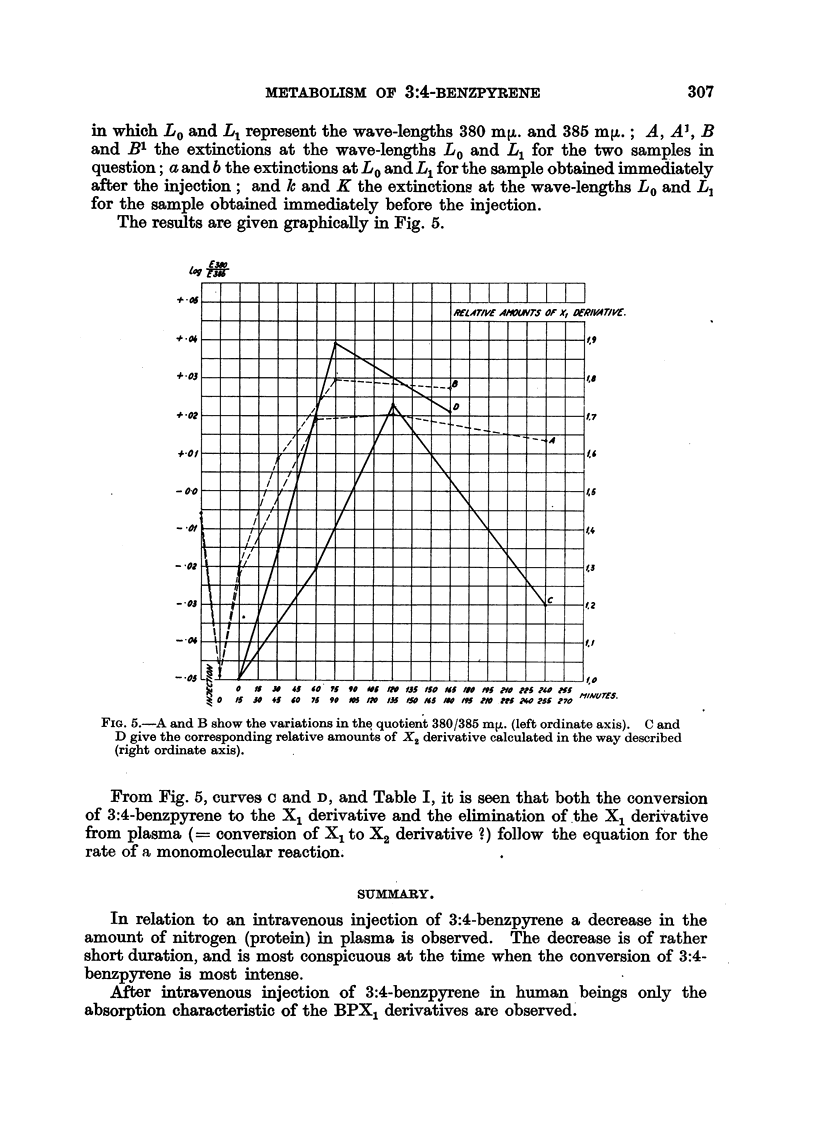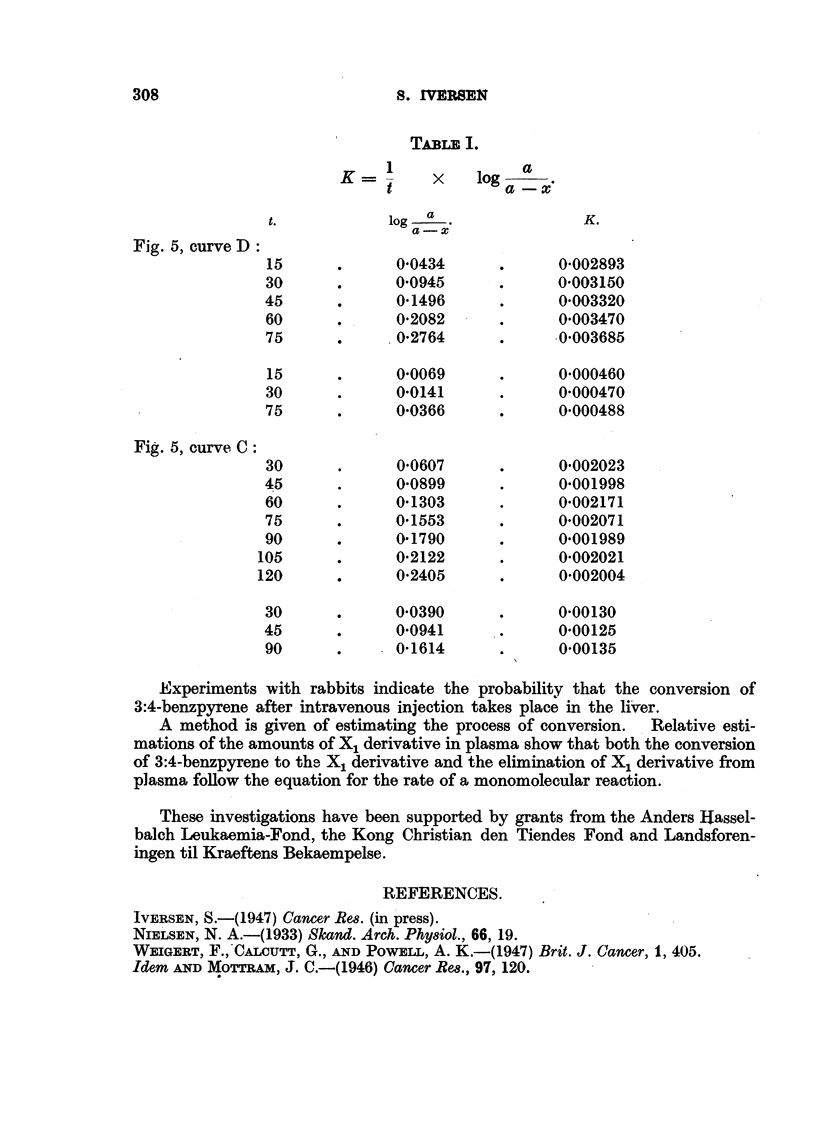# The Metabolism of 3:4-Benzpyrene in Human Beings after Intravenous Injection

**DOI:** 10.1038/bjc.1948.36

**Published:** 1948-09

**Authors:** S. Iversen


### THE METABOLISM OF 3:4-BENZPYRENE IN HUMAN BEINGS AFTER INTRAVENOUS INJECTION.

S. IVERSEN.*

*From the Institute of Pathological Anatomy, University of Copenhagen, Denmark.*

Received for publication June 11, 1948.

EARLIER investigations by Weigert and Mottram (1946) have shown that 3:4-benzpyrene after administration to the animal organism is metabolized to at least four different derivatives, labelled by Weigert and Mottram as $BPX_1$, $BPX_2$, $BPF_1$ $BPF_2$. In a later investigation Weigert, Calcutt and Powell showed that after application of 3:4-benzypyrene to the skin of mice only the presence of $BPX_2$ was detected.

That a similarity exists between the metabolism of 3:4-benzpyrene in animals and human beings (patients suffering from leukaemia) has already been shown (Iversen, 1947).

The following investigation shows that the intravenous injection of 3:4-benzpyrene in human beings has an effect upon the amount of nitrogen in plasma, and that only—or mainly—the $BPX_1$ derivatives are found in plasma.

### MATERIAL AND METHODS.

One part of 3:4-benzpyrene was fused with four parts of Postonal (a polymerization product of ethylenoxide), and carbon-dioxide free distilled water was added to the melted mixture, which was then shaken vigorously. This emulsion was stable for 3–4 hours. The diameter of the particles of the emulsion was between 6 to 15$\mu$.

Immediately after the emulsion was prepared it was injected intravenously in patients suffering from myeloid or lymphatic leukaemia. The doses used were 300, 500 and 800 mg. 3:4-benzpyrene in 30, 40 and 80 c.c. emulsion.

Blood samples (one part 3·8 per cent sodium-citrate solution plus 9 parts blood) were taken from a cubital vein immediately before, immediately after and at various times after the injection. The refractive index determined in sodium light with an Abbe's refractometer and the content of nitrogen by means of Kjeldahl analysis were determined in plasma obtained after centrifuging the blood samples for half an hour at 2000 r.p.m.

Five c.c. ethanol was added to 2 c.c. plasma, and after centrifuging for 15 minutes at 2000 r.p.m., the alcoholic solution was poured into measure glasses and the volume made up to 7 c.c. with ethanol, whereafter the absorption of the alcoholic solution was measured on a Beckmann photo-electric spectrophotometer.

After the precipitate was dried to constant weight the amount of nitrogen in the precipitate was determined by Kjeldahl analysis.

* Holder of a scholarship from the Lady Tata Memorial Trust.

FIG. 1.—A and B show the amount in mg. of dried precipitate in plasma from different blood samples obtained at various times before and after the injection. C and D show the variation of the refractive index of plasma in relation to the injection. E and F show the amount of nitrogen in (475 mm.$^3$) plasma.

Fig. 1, curves A and B, shows a distinct decrease in the weight of precipitate which is most conspicuous at approximately 15 minutes after the injection.

In curves C and D also the decrease is greatest at approximately 15 minutes after the injection.

Curves E and F show the same fluctuations as in A, B, C and D.

FIG. 2.—Absorption curves of plasma extracted with ethanol before and after intravenous injection of 3:4-benzpyrene.

In order to make sure that the emulsifier (Postonal) itself had no effect on the amount of nitrogen in plasma, 8 g. of Postonal, which is more than twice the amount ordinarily used for the benzpyrene emulsions, were injected intravenously into a patient. No changes in the plasma as described above were noticed.

Fig. 2 shows that the maxima of absorption characteristic of 3:4-benzpyrene are, little by little, altered to maxima of absorption characteristic of the metabolites labelled as $BPX_1$. The differences in position on the ordinate axis are seen to correspond with the fluctuations in the amount of nitrogen in plasma, which may mean that the fraction of alcohol-soluble substances varies correspondingly.

The results of Kjeldahl analysis on the precipitates showed that there was no noticeable difference between the percentage of nitrogen in the different samples of precipitate, which may mean that the composition of protein in plasma does not alter considerably in relation to an intravenous injection of 3:4-benzpyrene, or that it varies so little that it has been impossible, with the technique used, to determine the difference.

It would be possible to obtain a quantitative estimation of the proportion between the amount of unchanged benzpyrene and changed benzpyrene, and thus get an expression for the speed with which the metabolism (or detoxication ?) of 3:4-benzpyrene takes place by determining the proportions between the extinction at 380 mμ. (maximum for $X_1$ derivative) and at 385 mμ. (maximum for 3:4-benzpyrene).

The quotient $\frac{\text{E } 380 \text{ m}\mu.}{\text{E } 385 \text{ m}\mu.}$ for benzpyrene in 10 single determinations was shown to be 0·8599. The same quotient for the $X_1$ derivative is unknown and could not be determined, as it was not possible to obtain the $X_1$ derivative in a pure form, but the quotient must be higher than 1·00, as the absorption of the $X_1$ derivative is more intense at 380 mμ. than at 385 mμ.

The quotients obtained from alcohol-extracted plasma at different times before and after the injection are shown in Fig. 1, curves G and H. The curves show that the concentration of unchanged benzpyrene rapidly decreases, whereas the concentration of changed benzpyrene ($X_1$ derivative) rapidly increases. The curves further show that the time at which the rate of conversion is highest nearly coincides with the time at which the decrease in nitrogen-(protein-) content of the plasma is most conspicuous. Additionally the curves indicate that after the maximum concentration of changed hydrocarbon has been reached, elimination takes place at a constant speed.

That the conversion may take place in the liver after an intravenous injection is an obvious consideration, and the following experiment would indicate that this is the case in rabbits:

Blood is transfused through a living rabbit liver (Nielsen, 1933), and to the circulating blood is added 10 mg. 3:4-benzpyrene emulsified in the usual way; samples of blood are taken before and at various times after the injection. Fig. 3 shows, as in the case of human beings, that the absorption maxima of 3:4-benzpyrene are altered gradually to the maxima of absorption characteristic of the $X_1$ derivatives. That these curves appear higher and higher on the ordinate axis may be accounted for by the fact that it is not possible for the system to excrete. The variations in the quotient 380/385 mμ. from two such experiments with rabbits are seen in Fig. 4, curves D and E. The quotients for the alcohol extracted livers were 1·10 and 1·02. The fact that these quotients are higher than in the sera strongly supports the theory that the conversion of the hydrocarbon takes place in the liver.

Fig. 4, curve C, shows the variations of the quotient 380/385 mμ. for alcohol-extracted plasma from a patient suffering from lymphatic leukaemia. This curve has a noticeably different course from the quotient curve obtained from myeloid leukaemia, which may mean a slower rate of conversion, in the case of lymphatic leukaemia. Whether this difference in the rate of conversion of 3:4-benzpyrene is due to differences in the individuals themselves, to differences in the amount of benzpyrene injected, or to a difference between myeloid and lymphatic leukaemia, it is not yet possible to decide, since the material on lymphatic leukaemia has been too limited to permit definite conclusions. Fig. 4, curve C, indicates clearly that the conversion takes place at a markedly lower speed than in the other instances mentioned. In this connection it is an interesting phenomenon that it seems easier to influence a lymphatic leukaemia by

FIG. 3.—Absorption curves of the alcohol-extracted rabbit sera. (Defibrinated blood was nsed in the system.)

intravenous injections of polycyclic aromatic hydrocarbons than to influence a myeloid leukaemia (J. Engelbreth-Holm, private communication).

There are some observations which indicate that the metabolism of 3:4-benzpyrene is accompanied by changes in the metabolism of the protein. Thus it is a common reaction in all patients, after intravenous injection of benzpyrene (or other carcinogenic hydrocarbons), to get an attack of high temperature (40–41° C.), which appears approximately 1–2 hours after the injection and lasts for 4–6 hours. One of the patients, after an injection of 500 mg. benzpyrene, preceded by four earlier injections, sustained a severe attack of (protein ?) shock, which appeared 4–5 hours after the injection and lasted for 2–3 hours. It ought perhaps to be mentioned in this connection that the patients during, or immediately after, but never later than half an hour after the injection, sometimes got slight attacks of pulmonary infarcts.

Whether one considers the changes in the amount of nitrogen (protein) in plasma as attached to the metabolism of 3:4-benzpyrene or as bearing no relation

FIG. 4.—A and B = Myeloid leukaemia. C = Lymphatic leukaemia. D and E = Rabbit.

to the conversion of 3:4-benzpyrene, but accompanying the intravenous injection of 3:4-benzpyrene in an apparently regular way, it is possible, if one considers the concentration of unchanged benzpyrene as being highest in the blood sample obtained immediately after the injection, to calculate the relative amounts of $X_1$ derivative of the different blood samples by using the following equation :

$$\frac{\text{conc. } X}{\text{conc. } Y} = \frac{\left(A_{L_0} - k_{L_0}\right) - \left(\frac{a_{L_0} - k_{L_0}}{b_{L_1} - K_{L_1}} \cdot \left(A^1_{L_1} - K_{L_1}\right)\right)}{\left(B_{L_0} - {}_{L_0}\right) - \left(\frac{a_{L_0} - k_{L_0}}{b_{L_1} - K_{L_1}} \cdot \left(B^1_{L_1} - K_{L_1}\right)\right)}$$

in which $L_0$ and $L_1$ represent the wave-lengths 380 mμ. and 385 mμ.; $A$, $A^1$, $B$ and $B^1$ the extinctions at the wave-lengths $L_0$ and $L_1$ for the two samples in question; $a$ and $b$ the extinctions at $L_0$ and $L_1$ for the sample obtained immediately after the injection; and $k$ and $K$ the extinctions at the wave-lengths $L_0$ and $L_1$ for the sample obtained immediately before the injection.

The results are given graphically in Fig. 5.

FIG. 5.—A and B show the variations in the quotient 380/385 mμ. (left ordinate axis). C and D give the corresponding relative amounts of $X_2$ derivative calculated in the way described (right ordinate axis).

From Fig. 5, curves C and D, and Table I, it is seen that both the conversion of 3:4-benzpyrene to the $X_1$ derivative and the elimination of the $X_1$ derivative from plasma (= conversion of $X_1$ to $X_2$ derivative ?) follow the equation for the rate of a monomolecular reaction.

### SUMMARY.

In relation to an intravenous injection of 3:4-benzpyrene a decrease in the amount of nitrogen (protein) in plasma is observed. The decrease is of rather short duration, and is most conspicuous at the time when the conversion of 3:4-benzpyrene is most intense.

After intravenous injection of 3:4-benzpyrene in human beings only the absorption characteristic of the $BPX_1$ derivatives are observed.

TABLE I.

$$K = \frac{1}{t} \times \log \frac{a}{a - x}.$$

| | $t$. | $\log \frac{a}{a-x}$. | $K$. |
|---|---|---|---|
| Fig. 5, curve D : | | | |
| | 15 | 0·0434 | 0·002893 |
| | 30 | 0·0945 | 0·003150 |
| | 45 | 0·1496 | 0·003320 |
| | 60 | 0·2082 | 0·003470 |
| | 75 | 0·2764 | 0·003685 |
| | 15 | 0·0069 | 0·000460 |
| | 30 | 0·0141 | 0·000470 |
| | 75 | 0·0366 | 0·000488 |
| Fig. 5, curve C : | | | |
| | 30 | 0·0607 | 0·002023 |
| | 45 | 0·0899 | 0·001998 |
| | 60 | 0·1303 | 0·002171 |
| | 75 | 0·1553 | 0·002071 |
| | 90 | 0·1790 | 0·001989 |
| | 105 | 0·2122 | 0·002021 |
| | 120 | 0·2405 | 0·002004 |
| | 30 | 0·0390 | 0·00130 |
| | 45 | 0·0941 | 0·00125 |
| | 90 | 0·1614 | 0·00135 |

Experiments with rabbits indicate the probability that the conversion of 3:4-benzpyrene after intravenous injection takes place in the liver.

A method is given of estimating the process of conversion. Relative estimations of the amounts of $X_1$ derivative in plasma show that both the conversion of 3:4-benzpyrene to the $X_1$ derivative and the elimination of $X_1$ derivative from plasma follow the equation for the rate of a monomolecular reaction.

These investigations have been supported by grants from the Anders Hasselbalch Leukaemia-Fond, the Kong Christian den Tiendes Fond and Landsforeningen til Kraeftens Bekaempelse.

### REFERENCES.

IVERSEN, S.—(1947) *Cancer Res.* (in press).
NIELSEN, N. A.—(1933) *Skand. Arch. Physiol.*, **66**, 19.
WEIGERT, F., CALCUTT, G., AND POWELL, A. K.—(1947) *Brit. J. Cancer*, 1, 405.
*Idem* AND MOTTRAM, J. C.—(1946) *Cancer Res.*, **97**, 120.